# Pursuing Orally Bioavailable Hepcidin Analogues via Cyclic *N*-Methylated Mini-Hepcidins

**DOI:** 10.3390/biomedicines9020164

**Published:** 2021-02-08

**Authors:** Daniela Goncalves Monteiro, Johannes W. A. van Dijk, Randy Aliyanto, Eileen Fung, Elizabeta Nemeth, Tomas Ganz, Johan Rosengren, Richard J. Clark

**Affiliations:** 1 School of Biomedical Sciences, Faculty of Medicine, The University of Queensland, Brisbane, QLD 4072, Australia; gm.daniela1@gmail.com (D.G.M.); janwillemvandijk1984@gmail.com (J.W.A.v.D.); randy_aliyanto@hotmail.com (R.A.); j.rosengren@uq.edu.au (J.R.); 2 David Geffen School of Medicine, University of California, Los Angeles, CA 90095, USA; efung@mednet.ucla.edu (E.F.); ENemeth@mednet.ucla.edu (E.N.); TGanz@mednet.ucla.edu (T.G.)

**Keywords:** hepcidin, ferroportin, cyclic peptides, *N*-methylation, oral bioavailability

## Abstract

The peptide hormone hepcidin is one of the key regulators of iron absorption, plasma iron levels, and tissue iron distribution. Hepcidin functions by binding to and inducing the internalisation and subsequent lysosomal degradation of ferroportin, which reduces both iron absorption in the gut and export of iron from storage to ultimately decrease systemic iron levels. The key interaction motif in hepcidin has been localised to the highly conserved N-terminal region, comprising the first nine amino acid residues, and has led to the development of mini-hepcidin analogs that induce ferroportin internalisation and have improved drug-like properties. In this work, we have investigated the use of head-to-tail cyclisation and *N*-methylation of mini-hepcidin as a strategy to increase oral bioavailability by reducing proteolytic degradation and enhancing membrane permeability. We found that backbone cyclisation and *N*-methylation was well-tolerated in the mini-hepcidin analogues, with the macrocylic analogues often surpassing their linear counterparts in potency. Both macrocyclisation and backbone *N*-methylation were found to improve the stability of the mini-hepcidins, however, there was no effect on membrane-permeabilizing activity.

## 1. Introduction

In vertebrates, the highly conserved hepatocyte-secreted peptide hormone hepcidin is the primary regulator of iron absorption, plasma iron levels, and tissue iron distribution [1]. Hepcidin functions by binding to and either occluding or inducing the internalisation and lysosomal degradation of its receptor, ferroportin (FPN) [2,3]. This reduces both iron absorption in the gut and export of iron from storage organs, ultimately lowering systemic iron levels. Hepcidin has also been found to be produced locally and perform important functions with physiological and pathological implications for organ homeostasis [4,5,6]. The paramount role of hepcidin in iron homeostasis makes it a promising target for the development of diagnostic and therapeutic tools. However, due to the undesirable pharmacokinetics of unmodified hepcidin, pharmacologically suitable mimics of this peptide hormone are being investigated for application in pathologies hallmarked by iron overload, such as hereditary haemochromatosis, β-thalassaemia, and sickle cell disease [7,8].

**Figure 1:**
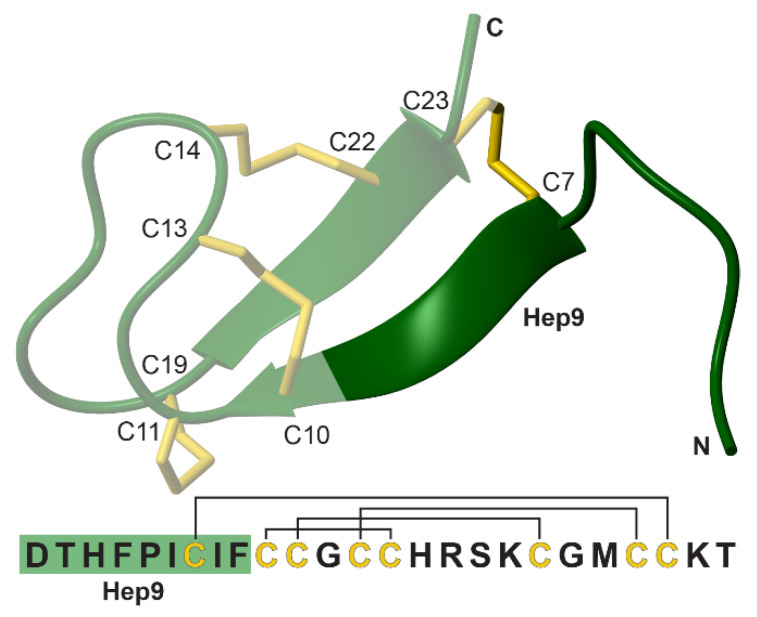
The sequence and structure of hepcidin (PDB ID 2KEF) [17]. The NH_2_-terminal region that interacts with ferroportin, hepcidin 9 (Hep9), is highlighted in dark green and the disulfide bonds in yellow. The disulfide connectivity is indicated on the sequence by black lines.

As a mature bioactive peptide, hepcidin is composed of 25 amino acids arranged in a bent β-hairpin that is cross-linked by four disulfide bonds (Cys^7^/Cys^23^, Cys^10^/Cys^13^, Cys^11^/Cys^19^ and Cys^14^/Cys^22^, Figure 1) [9]. A series of structural and functional studies have traced the bioactive epitope to the highly conserved N-terminal region comprised of the first 9 amino acid residues and, in particular, to the aromatic and hydrophobic patch arising from His^3^, Phe^4^, Ile^6^, and Phe^9^ [10,11]. This underpinned the development of mini-hepcidins, which are based on the N-terminal sequence of hepcidin (hepcidin 9, referred to as Hep9) [12]. Several mini-hepcidin derivatives have been extensively characterised, with studies showing the ability of these truncated peptides to reduce iron accumulation and lower serum iron levels in hepcidin-knockout and hereditary haemochromatosis mouse models [7,12]. Furthermore, hepcidin analogues or mimetics have entered trials for the treatment of iron-related disorders, including hereditary haemochromatosis, β-thalassaemia, sickle cell disease, and myelodysplastic syndrome, which supports the broad applicability and multifunctionality of this type of therapeutic replacement, either alone or as an adjuvant to phlebotomy, erythrocytapheresis or chelation therapies [13,14,15,16].

In this study we have investigated the approaches of head-to-tail cyclisation and *N*-methylation on mini-hepcidins with the ultimate goal of developing stable and potentially orally bioavailable hepcidin analogues. Both head-to-tail cyclisation and *N*-methylation have been used to reduce the conformational flexibility and solvation energies of bioactive peptides, thereby increasing resistance towards proteolytic degradation and enhancing membrane permeability [18,19,20,21]. Head-to-tail cyclisation not only shields both the N- and C-termini of a lead peptide, but also imparts a level of structural rigidity that further contributes to improved resistance towards enzyme-catalysed degradation, and often directs the peptide into conformations that are more favourable for diffusion across membranes [22]. Moreover, the additional constraint may even minimise the potential entropic cost of binding, resulting in increased potency.

Similarly to macrocyclisation, backbone *N*-methylation has also been found to enhance the affinity and refine the selectivity of peptides towards their binding partners [23,24]. Furthermore, *N*-methylation has been applied to peptide leads in ways that improve their oral bioavailability without significantly impacting their bioactivity [25]. Backbone *N*-methylation is thought to affect peptide conformation and hydrogen bonding potential which is, in turn, expected to influence membrane permeability [26].

Here, we have designed a series of backbone cyclic and *N*-methylated mini-hepcidin analogues and evaluated the effects of these modifications on activity, stability and membrane permeability. The results show that both head-to-tail cyclisation and backbone *N*-methylation are well tolerated by the mini-hepcidin lead, Hep9. Overall, the biological activity of the series is comparable to that of Hep9, with macrocyclic analogues often surpassing their linear counterparts in potency. Despite being active and stable, no enhancement in membrane permeability was observed for macrocyclic *N*-methylated analogues.

## 2. Experimental Section

### 2.1. General Methods

Reverse phase high performance liquid chromatography (RP-HPLC) was performed using a Shimadzu Prominence equipped with a Grace^TM^ Vydac^®^ C_18_, 250 × 21.2 mm preparative column or with a Grace^TM^ Vydac^®^ C_18_, 250 × 10 mm, semi-preparative column. Purifications involved a mobile phase of 0.05% (*v*/*v*) TFA (trifluoroacetic acid) in Milli-Q^®^ water (buffer A) mixed with 0.05% (*v*/*v*) TFA and 90% (*v*/*v*) ACN (acetonitrile) in Milli-Q^®^ water (buffer B) over a 0–80% buffer B gradient. Analytical RP-HPLC was performed using a Shimadzu Prominence equipped with a Grace^TM^ Vydac^®^ 218TP^TM^ C_18_, 150 × 2.1 mm, 5 µm, column.

Mass spectrometry (MS) was performed using an ABSciex API 2000^TM^ coupled to an Agilent 1260 Infinity, and analytical liquid chromatography mass spectroscopy (LC-MS) was performed using the same system equipped with a Kinetex^®^ 2.6 µm C_18_ 100 Å, 50 × 2.1 mm LC column. Separations involved a mobile phase of 0.1% (*v*/*v*) FA (formic acid) in Milli-Q^®^ water (solvent A) mixed with 0.1% (*v*/*v*) FA and 90% (*v*/*v*) ACN in Milli-Q^®^ water (solvent B). The separation method comprised a 5–95% solvent B gradient (0–6 min), 95% solvent B phase (6–8 min), 95–5% solvent B gradient (8–9 min), and an equilibration phase at 5% solvent B (9–12 min).

All ^1^H NMR spectra were recorded in H_2_O/D_2_O (90:10, pH ~4) on a Bruker Avance 600 MHz spectrometer. Spectra were assigned using CCPNMR Analysis [27].

### 2.2. Peptide Synthesis

Hepcidin analogues were synthesised using either manual or automated Fmoc (9-fluorenylmethyloxycarbonyl)-based solid-phase peptide synthesis (SPPS). For manual synthesis, peptides were assembled on 2-chlorotrityl chloride (2-CTC) resin by manual Fmoc (9-fluorenylmethyloxycarbonyl)-based solid-phase peptide synthesis (SPPS) using HATU as a coupling reagent (Table 1 and Table S1) [28]. Resin loading was achieved using the standard protocol for loading trityl-based linkers [28]. Subsequent couplings were carried out by adding Fmoc amino acids (4 equiv.), 1-[Bis (dimethylamino)methylene]-1H-1,2,3-triazolo[4,5-b]pyridinium 3-oxid hexafluorophosphate (HATU, 4 equiv.), and N, N-diisopropylethylamine (DIPEA, 4 equiv.) dissolved in *N,N*-dimethylformamide (DMF, up to 0.2 M with respect to the amino acid) to the resin and reacting for 1 to 2 h. Fmoc deprotection was achieved after each coupling using 20% (*v*/*v*) piperidine in DMF (2 × 5 mL, 5 min each). Coupling reactions were monitored using the ninhydrin test and double couplings were carried out for most residues, in particular those following *N*-methylated amino acids [28]. Cleavage of the protected peptide was accomplished with 1% (*v*/*v*) TFA in DCM (10 × 10 mL, 1–2 min each), which was followed by rotary evaporation of the filtrate and precipitation of the peptide with Milli-Q^®^ water. The precipitate was left to stand for at least 30 min on ice, after which it was filtered using a frit fitted syringe, dissolved in buffer B and lyophilized.

For automated peptide synthesis, mini-hepcidin analogues were synthesised on a Biotage^®^ Initiator+ Alstra^TM^ peptide synthesiser using Fmoc amino acids, Wang resin manually pre-loaded with Fmoc-Phe using the symmetrical anhydride protocol, and double couplings for all β–branched amino acids and those following *N*-methylated residues [28]. Amino acid residues were coupled using the in situ microwave-assisted HATU coupling protocol for Fmoc chemistry [28]. Fmoc amino acids (4 equiv.), HATU (4 equiv.), and DIPEA (4 equiv.) were dissolved in DMF (up to 0.2 M with respect to the amino acid), added to the resin and reacted for 5 min at 75 °C (standard coupling) or 30 min at 40 °C (coupling of histidine, cysteine and any *N*-methylated amino acids). Fmoc deprotection was carried out using 20% (*v*/*v*) piperidine in DMF (2 × 5 mL, 1 + 8 min). Once the coupling of all amino acids was completed, the resin was dried and, after cleavage for 2 h with a solution of TFA:H_2_O:TIPS:DODT (92.5:2.5:2.5:2.5), the filtrate was collected, evaporated, and the crude peptide precipitated with Milli-Q^®^ water. The precipitate was then filtered, dissolved in a mixture of buffer A and B, and lyophilised. Purification was carried out using a preparative RP-HPLC column and a 0.7% gradient of buffer B in buffer A. Fractions containing the linear peptide (>75%) were collected. When purity <95%, the fractions collected were repurified on a semi-preparative RP-HPLC column with a 0.6% gradient of buffer B in buffer A.

Peptide cyclisation was carried out overnight using PyBOP^®^ ((Benzotriazol-1-yloxy)tripyrrolidinophosphonium hexafluorophosphate, 5 equiv.) and DIPEA (15 equiv.) in a DMF solution of 10 mM of protected crude peptide. The reaction was quenched with Milli-Q^®^ water and the precipitated peptide was filtered, dissolved in a mixture of buffer A and buffer B, and lyophilised.

Final deprotection, when required, for linear or cyclic peptides, was achieved by treatment with a solution of TFA:H_2_O:TIPS:DODT (92.5:2.5:2.5:2.5) (trifluoroacetic acid, water, triisopropylsilane, and 3,6-dioxa-1,8-octane-dithiol) for 15 to 20 min, followed by solvent evaporation and loading onto a preparative RP-HPLC column as a solution of approximately 20% (*v*/*v*) buffer B in buffer A. A 1% gradient of buffer B was applied and fractions containing the cyclic peptide (>80%) were collected.

### 2.3. In Vitro Peptide Bioactivity Assay

HEK293:T-REx^TM^-FPN-GFP cells, described previously [29], were cultured in T75 flasks containing FPN-GFP media (Dulbecco’s Modified Eagle’s Medium (DMEM) containing high glucose, GlutaMAX^TM^ supplement, hydroxyethyl piperazineethanesulfonic acid (HEPES), 10% tetracycline screened foetal bovine serum (FBS), 1% penicillin/streptomycin, and 100 μg/mL of hygromycin) and maintained at 37 °C in a humidified atmosphere of 5% CO_2_.

**Figure 2:**
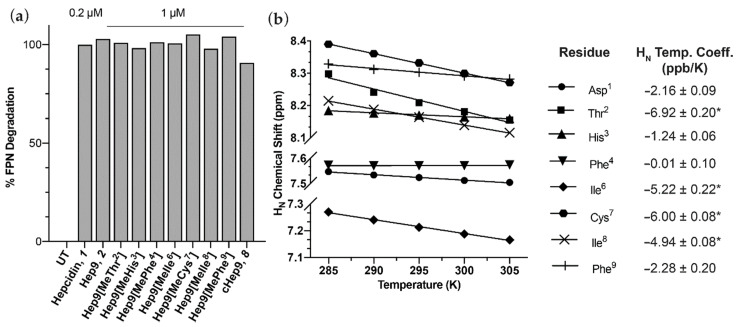
(**a**) Bioactivity of mono *N*-methylated mini-hepcidins. FPN-GFP expression was induced with doxycycline (50 ng/mL) for 24 h, and HEK293:T-RExTM-FPN-GFP cells were then treated with the respective peptides for another 24 h, after which FPN-GFP expression was quantitated by flow cytometry. (**b**) Temperature dependence of H_N_ chemical shifts for cHep9. H_N_ temperature coefficients marked with “*” are <−4.6 ppb/K.

HEK293:T-REx^TM^-FPN-GFP cells were plated in FPN-GFP media in the presence of 20 µM FAC (ferric ammonium citrate = ammonium iron (III) citrate) and induced on the following day with 50 ng/mL of doxycycline hydrochloride. After 24 h, doxycycline hydrochloride was washed off with PBS (phosphate buffered saline) and the cells were treated with peptides for another 24 h. Cells were then trypsinised, centrifuged (5 min at 1000 rpm), and resuspended in PBS at approximately 1 × 10^4^ cell/µL. The intensity of green fluorescence was measured by flow cytometry performed on a Becton Dickinson LSR Fortessa-X-20. Cells not induced with doxycycline hydrochloride were used to establish a gate to exclude background fluorescence, and cells induced to express FPN-GFP but not treated with any peptides were used as the positive control. Each peptide treatment was repeated independently at least three times except for the initial screening of the mono *N*-methylated analogues (Figure 2), which was only performed once. The results were expressed as the % of the reduction in fluorescence with respect to hepcidin-treated cells according to the following formula: % FPN Degradation = (1 − (average *F_Hepc_* − *F_χ_*)/(average *F_Hepc_* − average *F_untreated_*)) × 100, where *F* corresponds to the GFP gate median, and χ to the peptide analysed.

### 2.4. Serum Stability Assay

Human male serum was centrifuged at 20,000 g for 10 min and the clear part of the supernatant was then diluted to 25% (*v*/*v*) with Milli-Q^®^ water and incubated at 37 °C for 15 min. Each peptide sample was dissolved in triplicate at a final concentration of 20 μM in pre-incubated 25% serum, or Milli-Q^®^ water as a control. Aliquots of 100 μL were removed at different time points and quenched with 100 μL of 15% trichloroacetic acid (TCA), followed by incubation on ice for 30 min and centrifugation at 14,000 g for 5 min. Supernatant was then removed and transferred into vials for liquid chromatography mass spectrometry (LC-MS) analysis (50 μL injection and multiple reaction monitoring (MRM) protocol, Table S2).

### 2.5. Parallel Artificial Membrane Permeability Assay (PAMPA)

A Corning^®^ Gentest^TM^ 96-well microplate pre-coated with artificial phospholipids (0.4 μm polyvinylidene fluoride (PVDF) membrane) was used. Peptide samples, 300 μL of 50 μM peptide solution prepared in 5% (*v*/*v*) dimethyl sulfoxide (DMSO) in Hank’s balanced salt solution (HBSS), were added to the donor wells (bottom plate) and 200 μL of buffer (5% (*v*/*v*) DMSO in HBSS) were added to the acceptor wells (top plate). The acceptor plate was then lowered onto the donor plate, the lid was placed, and the assay plate system was subsequently wrapped with foil and incubated at room temperature for 5 h.

Peptide concentration from acceptor and donor wells was measured by LC-MS using positive ion mode and a multiple reaction monitoring (MRM) protocol (Table S2). As the concentration differences between acceptor and donor wells are significant, a dilution series of each peptide sample was prepared and injected into the LC-MS along with a peptide standard (HFE control, EDNSTSGFWRYGYDG, 1752.77 g mol^−1^) to generate a standard curve of area ratio of peptide sample to peptide standard versus peptide sample concentration for quantification. Samples from donor, 5–10 μL and acceptor wells, 40–100 μL were injected into the LC-MS and the analyte peak areas were recorded. The percentage permeability was calculated relative to the equilibrium concentration (*C_equil_*), and the permeability (*P_e_*, cm s^−1^) and mass retention (*R*, %) were calculated according to the following formulas: *C_equil_* = (*C_D_(t)* × *V_D_ + C_A_(t)* × *V_A_*)/*V_D_ + V_A_*, *P_e_* = −(ln(1 − *C_A_(t)*/*C_equil_*)/*A* × (1/*V_D_* + 1/*V_A_*) × *t*)), and *R* = 1 − (*C_D_(t)* × *V_D_* + *C_A_(t)* × *V_A_*)/(*C_0_* × *V_D_*); where *C_0_* corresponds to the initial compound concentration in the donor well (M), *C_D_(t)* and *C_A_(t)* to the compound concentration in the donor and acceptor wells at time *t* (min), *V_D_* and *V_A_* to the volume of the donor and acceptor wells (0.3 mL and 0.2 mL, respectively), *A* to the filter area (0.3 cm^2^), and *t* to the incubation time (18,000 s) [26].

## 3. Results

### 3.1. Peptide Design, Synthesis and Biological Activity

In the design of our first generation of mini-hepcidin analogues, we investigated if single modifications to the sequence could be introduced without affecting biological activity. Therefore, we synthesised a set of peptides containing a single *N*-methylation at each residue and a head-to-tail backbone cyclic analogue.

**Figure 3:**
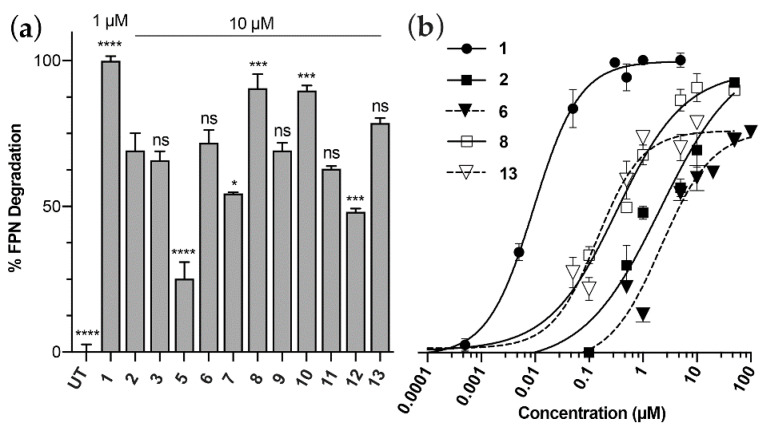
Bioactivity of a series of linear, cyclic and *N*-methylated mini-hepcidins. FPN-GFP expression was induced with doxycycline (50 ng/mL) for 24 h, and HEK293:T-RExTM-FPN-GFP cells were then treated with the respective peptides for another 24 h, after which FPN-GFP expression was quantitated by flow cytometry. Error bars indicate SEM, n ≥ 3. (**a**) Bioactivity scan of the peptide library. Significance is shown with respect to Hep9, ***2***. GP: 0.1234 (ns), 0.0332 (*), 0.0002 (***), <0.0001 (****) using one-way ANOVA with Dunnett’s test. (**b**) Dose-response studies comparing hepcidin **1**;Hep9, ***2***; cHep9, ***8***; and the most potent *N*-methylated analogues, Hep9[Ser^1^, MeThr^2^, MeIle^8^] **6**, and cHep9[Ser^1^, MeThr^2^, MeIle^8^] **13**.

Peptide bioactivity was measured using HEK293:T-REx^TM^-FPN-GFP cells stably expressing a GFP-tagged ferroportin (FPN) construct that was treated overnight with different concentrations of hepcidin or analogues. FPN-GFP expression and FPN-GFP internalisation and degradation in response to hepcidin treatment were confirmed by confocal microscopy, and the % of GFP fluorescence was then analysed by flow cytometry and expressed as a % FPN degradation. All analogues efficiently induced degradation of FPN-GFP in vitro at 1 μM and had activity that was comparable to unmodified Hep9, **2**, (Figure 2a). Interestingly, head-to-tail backbone cyclisation appears to constrain the mini-hepcidin lead in a way that facilitates ferroportin binding, with cHep9, ***8***, showing higher levels of activity than Hep9 at a concentration of 10 μM (**2** and **8**, Figure 3a). This promising result led us to design a second series of analogues that included multiple *N*-methylated residues with and without backbone cyclisation.

Recently, Wang, C. et al. have shown that amide proton temperature coefficients measured by NMR can be used to identify appropriate amides for *N*-methylation in short cyclic peptides, with the modified derivatives exhibiting improved solvation properties and enhanced membrane permeability [26]. Backbone amide protons with largely negative NMR temperature coefficients, Δδ_NH_/ΔT < −4.6 ppb/K, are water exposed, whilst those with less negative temperature coefficients, Δδ_NH_/ΔT ≥ −4.6 ppb/K, appear to be involved in stabilising intramolecular hydrogen bonding [30]. Therefore, we calculated the backbone amide proton temperature coefficients of cHep9 by measuring TOCSY NMR spectra at a series of temperatures and measuring change in chemical shift with temperature (Figure 2b). The temperature coefficients suggested that Thr^2^, Ile^6^, Cys^7^, and Ile^8^ are the most extensively solvated positions, with amide temperature coefficients ranging from −6.92 ppb/K to −4.94 ppb/K (Figure 2b).

In addition to the temperature coefficient analysis, we also undertook a serum stability analysis of Hep9, as *N*-methylation is an effective approach to protecting amide bonds from proteolytic cleavage. We identified the most vulnerable bonds to proteolytic cleavage as the N- and C-termini (which will be protected by backbone cyclisation), and the amide bonds between Pro^5^ and Ile^6^ and between Cys^7^ and Ile^8^ that would be protected by *N*-methylation of Ile^6^ and Ile^8^ (Figure S1). Based on these analyses, a series of mini-hepcidin agonists was designed comprising different combinations of *N*-methylated residues and backbone cyclisation (Table 1 and Table S1).

Our first step was to introduce multiple *N*-methylations at the identified positions within a linear Hep9 analogue. Complications were encountered in the synthesis of doubly and triply *N*-methylated mini-hepcidin analogues, which included difficulties with the on-resin *N*-methylation of cysteine, the incorporation of *N*-methylated amino acids, and the coupling of subsequent amino acid residues onto the sterically hindered *N*-methylated amine functional group. To overcome these hindrances, we used microwave-assisted synthesis, which has been reported to facilitate coupling of *N*-methylated amino acid residues [31]. We further modified our synthetic approach to employ Wang resin, which is more stable under microwave conditions. However, as we were unable to cleave a fully side-chain-protected peptide, which is required for backbone cyclisation, from Wang resin, we replaced the Asp^1^ with the functionally similar, but unreactive towards amine coupling, serine residue (Hep9[Ser^1^] **3**) to minimise any by-products during cyclisation. Previous studies have shown that changes in residue 1 of hepcidin have little effect on biological activity [10,11], and this was confirmed using the FPN-GFP internalisation assay (Table 1).

Using both of these synthetic strategies, we produced a set of di-*N*-methylated mini-hepcidin analogues (**4**–**6**, Table 1), and a tri-*N*-methylated derivative, Hep9[Ser^1^, MeThr^2^, MeIle^6^, MeIle^8^] **7**. Regrettably, the *N*-methylated cysteine analogues could only be produced at low yield and purity (Table S1).

The results from the FPN-GFP-based assay showed that analogues with multiple backbone *N*-methylation can efficiently bind and down-regulate ferroportin expression in vitro (**4**–**7**, Figure 3a). Of the three linear *N*-methylated and Asp^1^Ser variants shown: Hep9[Ser^1^, MeThr^2^, MeIle^6^] **5**; Hep9[Ser^1^, MeThr^2^, MeIle^8^] **6**; and Hep9[Ser^1^, MeThr^2^, MeIle^6^, MeIle^8^] **7**, the most potent appears to be Hep9[Ser^1^, MeThr^2^, MeIle^8^], **6**, closely followed by Hep9[Ser^1^, MeThr^2^, MeIle^6^, MeIle^8^] **7**. However, considering the challenges faced in the synthesis of the tri-*N*-methylated derivative, we chose to restrict the synthesis of cyclic Hep9 analogues to those with only two residues *N*-methylated.

We synthesised three cyclic di-*N*-methylated Hep9 analogues and found that these peptides were consistently more potent than their linear counterparts (**11–13**, Figure 3a,b). The bioactivity of the cyclic *N*-methylated derivative cHep9[Ser^1^, MeThr^2^, MeIle^8^] **13**, surpassed both cHep9[MeIle^6^, MeIle^8^] **12**, and cHep9[MePhe^4^, MePhe^9^] **11**, thereby becoming the most promising cyclic *N*-methylated lead.

Lastly, we produced two analogues of backbone cyclic Hep9. The first to see if a linker sequence connecting the amino and carboxyl termini within cHep9 (cHep9-Gly_4_ **10**) would favour the binding of macrocyclic analogues to the ferroportin receptor, as direct head-to-tail cyclisation might impose constraints that could limit the establishment of key binding interactions. In this instance, we found that cHep9-Gly_4_ has a slightly improved potency relative to cHep9.

### 3.2. Structural Comparison of Acyclic and Cyclic N-Methylated Mini-Hepcidins

Taking into account the results from the bioactivity assay, we then tried to further understand how the modifications introduced affected the overall arrangement of the mini-hepcidin derivatives relative to Hep9, cHep9, and the N-terminal region of wild-type hepcidin using NMR spectroscopy. The Hα secondary chemical shifts for each residue were calculated from the difference between assigned Hα chemical shifts and those of equivalent amino acids in a random coil peptide [27,32]. This is a commonly employed approach for deriving information on the structural organisation of peptides and making comparisons between related sequences [33].

**Figure 4:**
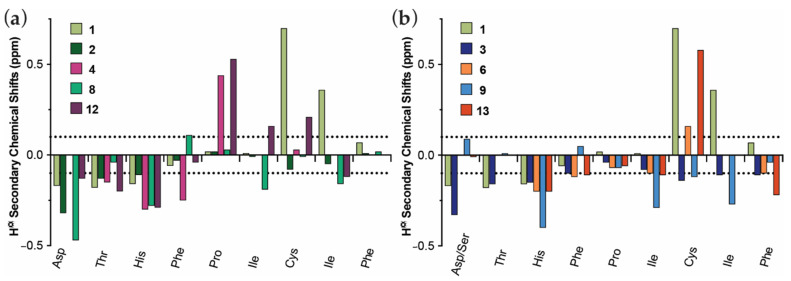
Comparison of the Hα secondary chemical shifts for the mini-hepcidin epitope (DTHFPICIF) for hepcidin and different hepcidin analogues. All ^1^H NMR spectra were recorded at 298 K on a Bruker Avance 600 MHz spectrometer. Spectra were assigned using CCPNMR Analysis [27]. (**a**) Comparison between hepcidin **1**, Hep9 **2**, Hep9[MeIle^6^, MeIle^8^] **4**, cHep9 **8,** and cHep9[MeIle^6^, MeIle^8^] **12**. (**b**) Comparison between hepcidin **1**, Hep9[Ser^1^] **3**, Hep9[Ser^1^, MeThr^2^, MeIle^8^] **6**, cHep9[Ser^1^] **9**, and cHep9[Ser^1^, MeThr^2^, MeIle^8^] **13**.

Hα secondary chemical shift values around 0 ppm (±0.1 ppm) typically correspond to unstructured regions, with stretches of positive (>0.1 ppm) and negative (<−0.1 ppm) values suggesting α-helical and β-sheet arrangements, respectively [33]. The structure of the N-terminus of native hepcidin is not well defined up to Ile^6^ [17]. The following amino acid residue, Cys^7^, is disulfide bonded to Cys^23^ and delineates the beginning of the first β-strand, which extends up to Cys^10^, in turn disulfide bonded to Cys^13^. As a result, Hep9, and any mini-hepcidin derivatives, in particular linearized versions, are expected to exhibit a large degree of conformational flexibility, as they lack the cystine network stabilising the bent β-hairpin characteristic of native hepcidin. As expected, the Hα secondary chemical shift values for all residues in both Hep9 **2**, and Hep9[Ser^1^] **3**, are approximately 0 ppm (Figure 4a,b). Conversely, for both cHep9 **8**, and cHep9[Ser^1^] **9**, the Hα secondary chemical shifts deviate from 0 ppm, with negative Hα secondary chemical shift values (−0.4 to −0.3 ppm) for His^3^, Ile^6^, and Ile^8^ (Figure 4a,b).

Furthermore, taking into account the structural differences between hepcidin and the mini-hepcidin analogues, it is not surprising that the main chemical shift deviations observed between these are located towards the middle and last few residues of the sequence of Hep9 (Figure 4a,b). Notably, Cys^7^ experiences a nuclear deshielding effect in native hepcidin that leads to a largely positive Hα secondary chemical shift (0.73 ppm), even after taking into account the disulfide bonding effects in the random coil value. For the mini-hepcidin derivatives, the sulfhydryl functional group of Cys^7^ is found in the reduced state, generally resulting in lower Hα secondary chemical shifts that fall within ±0.2 ppm. The only exception to this is Hep9[Ser^1^, MeThr^2^, MeIle^8^], ***6***, for which the Hα secondary chemical shift for Cys^7^ was calculated to be 0.58 ppm. Considering that this was found to be one of the most potent analogues, this may be indicative that the combination of the chemical modifications introduced, head-to-tail cyclisation and *N*-methylation at Thr^2^ and Ile^8^, may favour the adoption of the active conformation of the N-terminus of hepcidin.

It has been previously noted that *N*-methylation affects not only the conformation of the amino acid to which the methyl group is attached, but also that of the preceding residue [34]. This was also observed for this series of analogues, where both Hep9[MeIle^6^, MeIle^8^] **4**, and cHep9[MeIle^6^, MeIle^8^] **12**, showed a marked increase in the Hα secondary chemical shift of Pro^5^, which precedes the *N*-methylated Ile^6^ residue. Similarly, increases in the Hα secondary chemical shift of Cys^7^ were also noted for cHep9[MeIle^6^, MeIle^8^] **12**, and both Hep9[Ser^1^, MeThr^2^, MeIle^8^] **6**, and cHep9[Ser^1^, MeThr^2^, MeIle^8^] **13**, where Cys^7^ precedes the *N*-methylated Ile^8^ residue (Figure 4a,b). However, this downfield shift is not as large for cHep9[MeIle^6^, MeIle^8^] **12**, nor Hep9[Ser^1^, MeThr^2^, MeIle^8^] **6**, suggesting that *N*-methylation at Ile^8^ and macrocyclisation alone are not sufficient to induce the degree of deshielding experienced by Cys^7^ in cHep9[Ser^1^, MeThr^2^, MeIle^8^] **13**.

In summary, larger secondary shift deviations were calculated for both cyclic and *N*-methylated mini-hepcidins, suggesting these might adopt a more defined structure in solution relative to linear and non-*N*-methylated analogues. In addition, for both cHep9 **8**, and cHep9[Ser^1^, MeThr^2^, MeIle^8^] **13**, the added structural constraints appear to favour ferroportin binding.

### 3.3. cHep9 and N-Methylated Mini-Hepcidins Show Improved Serum Stability

The effect of these strategies on the stability of mini-hepcidin analogues was investigated by incubating selected derivatives in human male serum at 37 °C and collecting samples at various time points. The results were then quantitated by liquid chromatography-mass spectrometry (LC-MS). We were unable to determine the serum stability of all analogues as the recovery from serum and detection of the peptides was very low. Modification of sample preparation protocols, optimisation of LC/MS conditions, and reducing serum concentration to 25% were unable to improve yield/detection. We would predict that this poor recovery is due to high binding of peptides to serum proteins, which is consistent with native hepcidin, which binds to α-2-macroglobulin [35,36]. In agreement with previous peptide lead optimisation studies, both cyclisation and *N*-methylation were found to shield the peptide analogues from proteolytic degradation. In addition, when considered individually, it appears that *N*-methylation at key positions is more efficient than head-to-tail cyclisation, as the peptide half-life was found to increase from linear Hep9 **2**, ~3 min to cHep9 **8**, ~8 min and then again to Hep9[Ser^1^, MeThr^2^, MeIle^8^] **6**, ~12 min, as shown in Figure S1. Even with both N- and C-termini exposed, the latter is approximately four times more stable than Hep9, illustrating the significant effect of multiple *N*-methylation on stability.

### 3.4. Cell Permeability of Rationally Designed N-Methylated Mini-Hepcidins

**Figure 5:**
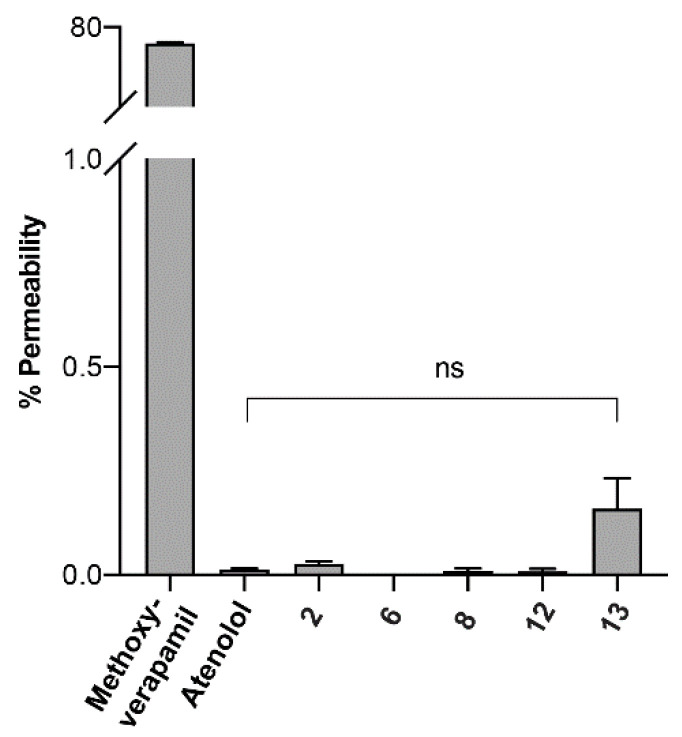
Membrane permeability of selected mini-hepcidins using PAMPA. Peptide samples, 300 μL of 50 μM peptide solution prepared in 5% (*v*/*v*) dimethyl sulfoxide (DMSO) in Hank’s balanced salt solution (HBSS) were added to the donor wells and 200 μL of buffer (5% (*v*/*v*) DMSO in HBSS) were added to the acceptor wells of a Corning^®^ Gentest^TM^ 96-well microplate pre-coated with artificial phospholipids. The acceptor plate was then lowered onto the donor plate and the assay plate system was incubated at room temperature for 5 h. Peptide concentration from acceptor and donor wells was measured by LC-MS using multiple reaction monitoring (MRM) protocol. The percentage permeability was calculated relative to the equilibrium concentration. Error bars indicate SEM, *n* = 3. Comparison between Hep9 **2**, Hep9[Ser^1^, MeThr^2^, MeIle^8^] **6**, cHep9 **8**, cHep9[MeIle^6^, MeIle^8^] **12**, and cHep9[Ser^1^, MeThr^2^, MeIle^8^] **13**. Methoxyverapamil and atenolol were used as controls for high and low permeability, respectively. ns: 0.1234.

It has been proposed that by enhancing the lipophilicity of peptides, the intestinal permeability could be improved by facilitating transcellular absorption (passive diffusion) [37]. Thus, our rationale was to improve the permeability of Hep9 ***2***, by increasing its lipophilicity via both cyclisation and multiple *N*-methylations. Accordingly, the most active analogues of the *N*-methylated series were assessed by the parallel artificial membrane permeation assay (PAMPA) and by the Caco-2 in vitro model. Peptide concentration from acceptor and donor wells was measured by liquid chromatography mass spectrometry (LC-MS) using a multiple reaction monitoring (MRM) protocol and a calibration curve generated by injecting a dilution series of each peptide sample in the presence of a peptide standard. Unfortunately, according to our findings in the PAMPA model, all the tested peptides were found to have very low percentages of permeability, as calculated relative to the equilibrium concentration (Figure 5). Cyclisation and *N*-methylation appear to facilitate the crossing of the artificial lipid membrane for cHep9[Ser^1^, MeThr^2^, MeIle^8^] **13**, but this enhancement was not statistically significant relative to atenolol, a marker for poor transcellular permeability, or Hep9 **2**, (Figure 5). Notably, all cyclic derivatives tested, as well as Hep9, were found to bind non-specifically to the plastic surfaces of the permeation assay, with percentages of mass retention of approximately 30–50%. Even though this was taken into account within the permeability formula, it considerably reduced the limit of detection of peptide by LC-MS. Preliminary results from a Caco-2 in vitro model were consistent with the PAMPA data (data not shown).

## 4. Discussion

As prototype modulators of biological systems, peptides have an astounding range of functional applications and provide a rich diversity of pharmacophore leads for the conception of innovative and safe diagnostic tools and therapeutic drugs [38]. The major obstacle in the development of peptide therapeutics is, however, the challenging path from a biologically active lead to an orally bioavailable drug [39,40]. Here, we have used two consonant strategies to improve the stability and membrane permeability of mini-hepcidins: head-to-tail cyclisation and backbone *N*-methylation.

Backbone *N*-methylation has been recognised as a particularly powerful tool to modulate the pharmacokinetic profile and biological activity of therapeutic peptides [41,42,43]. Similarly to others, we have been inspired by the cyclic and highly *N*-methylated transplantation drug cyclosporine A, which, in spite of violating all of the Lipinski’s rules for drug design, has an average (though variable) oral bioavailability of approximately 30%, which has been linked to the greatly increased lipophilicity of the peptide resultant from extensive *N*-methylation [44].

The series of cyclic *N*-methylated mini-hepcidin analogues was rationally designed using ^1^H NMR to predict amide solvent exposure and guide the positioning of methyl functional groups onto the most extensively solvated amides. Recent reports show that this method has successfully led to improvements in the membrane permeability of peptides by favouring the adoption of structural conformations that have greatly reduced energetic penalties for membrane partition [26]. Using this rational approach means that the number of peptides synthesised can be drastically curtailed from the 255 (2^8^ − 1, as proline cannot be *N*-methylated) possible *N*-methylated derivatives of Hep9 to 15 (2^4^ − 1). Furthermore, here, we have used this *N*-methylation strategy to protect the amide bonds of Hep9 that are the most vulnerable to proteolytic degradation, with the overall aim of developing a shielded *N*-methylated mini-hepcidin lead for oral delivery.

The results from the bioactivity analysis of singly *N*-methylated mini-hepcidins showed that maximal activity was maintained for the whole series and potency levels were comparable to those of Hep9. Interestingly, this remained true for most of the multiply *N*-methylated analogues tested, suggesting that ferroportin binding is quite tolerant to the addition of small hydrophobic functional groups to backbone amides.

Similarly, head-to-tail cyclisation appears to have no negative impact in the binding interaction with ferroportin and, in some cases, it seems to even favour the biologically active conformation relative to the linear counterparts. These promising results are well in agreement with the structural analysis carried out using Hα secondary chemical shifts, where cHep9 and, in particular, the most potent analogue of the series, cHep9[Ser^1^, MeThr^2^, MeIle^8^], more closely resemble the pattern of the binding epitope of native hepcidin.

Even though the serum stability of some of the mini-hepcidin derivatives could not be determined, a trend of increased resistance to proteolytic degradation was observed from Hep9 to cHep9 and doubly *N*-methylated Hep9, supporting the applicability of both strategies for the improvement of the pharmacokinetic properties of short peptide leads. Regrettably, testing of selected mini-hepcidins using the in vitro parallel artificial membrane permeation assay (PAMPA) suggests that the two most biologically active mini-hepcidin derivatives, cHep9[MeIle^6^, MeIle^8^] and cHep9[Ser^1^, MeThr^2^, MeIle^8^], are both poorly lipid permeable by passive diffusion. Preliminary results from the Caco-2 in vitro model for cHep9[Ser^1^, MeThr^2^, MeIle^8^] paralleled these observations, but these need to be confirmed. A caveat of this optimisation strategy is that *N*-methylation of extensively solvated backbone amides does not necessarily enhance membrane permeability. Other contributing factors include sequence composition and backbone conformation, which, in this case, may be limiting the permeability of these mini-hepcidin derivatives. Similarly, in a study of rational *N*-methylation of the externally-oriented backbone amide protons of a cyclic integrin peptide ligand, none of the seven analogues were found to readily cross lipid membranes [41]. Additional studies have also found that backbone *N*-methylation improved the intestinal permeability of a series of peptide analogues by facilitating paracellular transport in the aqueous media of the tight-junctions located between the enterocytes, rather than by enabling the transcellular route characteristic of lipophilic compounds [25].

Overall, these results suggest that head-to-tail cyclisation and *N*-methylation at key positions introduce structural features that allow and may even favour ferroportin binding. Therefore, the combination of such strategies seems propitious for peptide optimisation and development of potent hepcidin mimics that may, in the future, turn into orally bioavailable therapeutics. Even if multiple *N*-methylation and head-to-tail cyclisation prove ineffective on their own, a range of other approaches may be considered, including some of the innovative nano-technology-based delivery systems, which could facilitate target delivery and significantly reduce the therapeutic dosage.

## Figures and Tables

**Table 1:** Library of mini-hepcidin analogues tested for FPN degradation activity.

#	Peptide	Sequence ^1^	FPN Degradation % ± SEM ^2^	pEC50 ± SEM
**1**	Hepcidin	DTHFPICIFCCGCCHRSKCGMCCKT	100 ± 2.6	8.028 ± 0.081
**2**	Hep9	DTHFPICIF	69 ± 10	5.676 ± 0.296
**3**	Hep9[Ser^1^]	STHFPICIF	66 ± 5.3	6.487 ± 0.124
**4**	Hep9[MeIle^6^, MeIle^8^]	DTHFP[*N*-Me I]C[*N*-Me I]F	Not active	-
**5**	Hep9[Ser^1^, MeThr^2^, MeIle^6^]	S[*N*-Me T]HFP[*N*-Me I]CIF	25 ± 9.9	4.39 ± 0.982
**6**	Hep9[Ser^1^, MeThr^2^, MeIle^8^]	S[*N*-Me T]HFPIC[*N*-Me I]F	72 ± 7.7	5.663 ± 0.117
**7**	Hep9[Ser^1^, MeThr^2^, MeIle^6^, MeIle^8^]	S[*N*-Me T]HFP[*N*-Me I]C[*N*-Me I]F	54 ± 0.8	5.723 ± 0.117
**8**	cHep9	c(DTHFPICIF)	91 ± 8.2	6.487 ± 0.124
**9**	cHep9[Ser^1^]	c(STHFPICIF)	69 ± 4.4	6.575 ± 0.096
**10**	cHep9-Gly_4_	c(DTHFPICIFGGGG)	90 ± 2.8	6.800 ± 0.072
**11**	cHep9[MePhe^4^, MePhe^9^]	c(DTH[*N*-Me F]PICI[*N*-Me F])	63 ± 1.6	-
**12**	cHep9[MeIle^6^, MeIle^8^]	c(DTHFP[*N*-Me I]C[*N*-Me I]F)	48 ± 1.9	5.537 ± 0.162
**13**	cHep9[Ser^1^, MeThr^2^, MeIle^8^]	c(S[*N*-Me T]HFPIC[*N*-Me I]F)	79 ± 3.0	6.853 ± 0.129

^1^ “c” corresponds to cyclic peptides and “*N*-Me” corresponds to *N*-methylated. ^2^ FPN degradation induced by peptide (10 μM) normalised with respect to hepcidin (1 μM). *n* = 3.

## Data Availability

Not applicable.

## Supplementary Materials

The following are available online at https://www.mdpi.com/2227-9059/9/2/164/s1, Table S1: Library of mini-hepcidin analogues that could not be tested, Table S2: Liquid chromatography mass spectrometry (LC-MS) specifications for multiple reaction monitoring (MRM) protocols, Figure S1: Effect of macrocyclisation and *N*-methylation on the stability of selected mini-hepcidin derivatives.
