# Supplementary material for: Pursuing Orally Bioavailable Hepcidin Analogues via Cyclic N-Methylated Mini-Hepcidins
Source: Biomedicines. 2021 Feb 8;9(2):164. doi: 10.3390/biomedicines9020164 (PMC7915682; doi:10.3390/biomedicines9020164)
Supplement: Supplementary file 1 [file biomedicines-09-00164-s001.pdf]

Supplementary Information

Pursuing orally bioavailable hepcidin analogues via cyclic N-methylated mini-hepcidins

Daniela Goncalves Monteiro[1], Johannes W. A. van Dijk[1], Randy Aliyanto[1], Eileen Fung[2], Elizabeta Nemeth[2], Tomas Ganz[2], K. Johan Rosengren[1], Richard J. Clark[1*]

[1]*School of Biomedical Sciences, Faculty of Medicine and Biomedical Sciences, University of Queensland, Chancellors Pl, Brisbane, QLD 4072, Australia*

[2]*David Geffen School of Medicine, University of California, Los Angeles, Los Angeles, California, USA.*

*Corresponding author. Richard J. Clark

Tel.: +61 7 3365 1527

E-mail address: richard.clark@uq.edu.au

Table S1. Library of mini-hepcidin analogues that could not be tested.

#	Peptide	Sequence[1]	Calc. [M+H]+	Obs. [M+H]+	Yield[2]
**1**	Hepcidin	D T H F P I C I F C C G C C H R S K C G M C C K T	2789.42	[M+3H+] 930.5	5%
**2**	Hep9	D T H F P I C I F	1092.27	1092.5	20%
**3**	Hep9[Ser^1]	S T H F P I C I F	1064.26	1064.6	5-10%
**4**	Hep9[MeIle^6, MeIle^8]	D T H F P [*N*-Me I] C [*N*-Me I] F	1120.32	1120.4	5%
**5**	Hep9[Ser^1, MeThr^2, MeIle^6]	S [*N*-Me T] H F P [*N*-Me I] C I F	1092.31	1092.6	< 5%
**6**	Hep9[Ser^1, MeThr^2, MeIle^8]	S [*N*-Me T] H F P I C [*N*-Me I] F	1092.31	1092.8	< 5%
**7**	Hep9[Ser^1, MeThr^2, MeIle^6, MeIle^8]	S [*N*-Me T] H F P [*N*-Me I] C [*N*-Me I] F	1106.34	1106.5	< 3%
**8**	cHep9	c(D T H F P I C I F)	1074.26	1074.6	10%
**9**	cHep9[Ser^1]	c(S T H F P I C I F)	1046.24	1046.7	5%
**10**	cHep9-Gly$_4$	c(D T H F P I C I F G G G G)	1302.46	1302.8	5%
**11**	cHep9[MePhe^4, MePhe^9]	c(D T H [*N*-Me F] P I C I [*N*-Me F])	1102.31	1102.6	5%
**12**	cHep9[MeIle^6, MeIle^8]	c(D T H F P [*N*-Me I] C [*N*-Me I] F)	1102.31	1102.6	< 5%
**13**	cHep9[Ser^1, MeThr^2, MeIle^8]	c(S [*N*-Me T] H F P I C [*N*-Me I] F)	1074.30	1074.6	< 3%
**14**	Hep9[MeThr^2, MeCys^7]	D [*N*-Me T] H F P I [*N*-Me C] I F	1120.32	1120.8	Could not purify
**15**	Hep9[MeThr^2, MeIle^6, MeCys^7]	D [*N*-Me T] H F P [*N*-Me I] [*N*-Me C] I F	1134.35	-	Could not synthesise
**16**	Hep9[MeThr^2, MeCys^7, MeIle^8]	D [*N*-Me T] H F P I [*N*-Me C] [*N*-Me I] F	1134.35	-	Could not purify
**17**	Hep9[Ser^1, MeThr^2, MeCys^7]	S [*N*-Me T] H F P I [*N*-Me C] I F	1092.31	-	Could not synthesise
**18**	cHep9[MeThr^2, MeCys^7]	D [*N*-Me T] H F P I [*N*-Me C] I F	1102.31	1102.7	Could not purify
**19**	cHep9[MeThr^2, MeIle^6, MeCys^7]	D [*N*-Me T] H F P [*N*-Me I] [*N*-Me C] I F	1116.34	-	Could not synthesise

**20**	cHep9[MeThr[2], MeCys[7], MeIle[8]]	D [*N*-Me T] H F P I [*N*-Me C] [*N*-Me I] F	1116.34	1116.5	Could not purify
**21**	cHep9[Ser[1], MeThr[2], MeIle[6]]	S [*N*-Me T] H F P [*N*-Me I] C I F	1074.30	1074.6	Could not purify
**22**	cHep9[Ser[1], MeThr[2], MeIle[6], MeIle[8]]	S [*N*-Me T] H F P [*N*-Me I] C [*N*-Me I] F	1088.33	1088.6	Could not purify
**23**	cHep9[Ser[1], MeThr[2], MeCys[7]]	S [*N*-Me T] H F P I [*N*-Me C] I F	1074.30	-	Could not synthesise

[1]"c" corresponds to cyclic peptides and "*N*-Me" corresponds to *N*-methylated. [2]%Yield was calculated relative to crude product following HPLC purification.

Table S2. Liquid chromatography mass spectrometry (LC-MS) specifications for multiple reaction monitoring (MRM) protocols.

#	Compound	Obs. [M+xH$^+$]	MRM transitions	Retention time (min)
**-**	Atenolol	267.1	225.1<br>190.1<br>145.2	1.19
**-**	Methoxyverapamil	485.4	333.4<br>165.2<br>150.1	6.79
**2**	Hep 9	1092.7	501.1<br>354.2<br>336.0	6.54
**4**	Hep9[MeIle^6, MeIle^8]	1120.7	372.4<br>245.2<br>217.5	6.48
**6**	Hep9[Ser^1, MeThr^2, MeIle^8]	1092.0	314.4<br>285.5<br>203.3	6.48
**8**	cHep9	1074.7	314.2<br>216.9<br>183.2	6.96
**12**	cHep9[MeIle^6, MeIle^8]	1102.9	294.4<br>217.1<br>156.2	7.15
**13**	cHep9[Ser^1, MeThr^2, MeIle^8]	1074.5	314.3<br>216.8<br>209.1	6.98

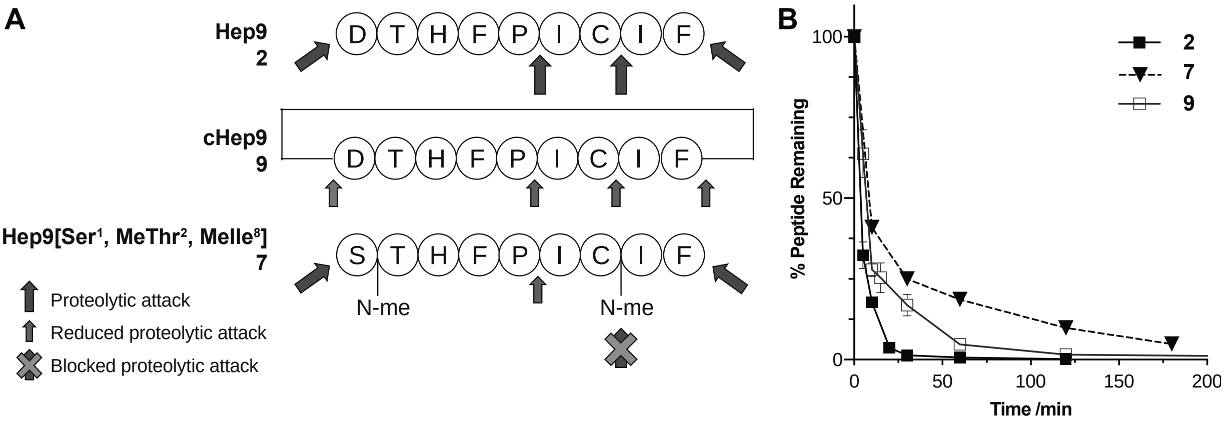

Figure S1. Effect of macrocyclisation and *N*-methylation on the stability of selected mini-hepcidin derivatives. **A**) Illustration of sites most susceptible to enzymatic attack in mini-hepcidin derivatives. **B**) Serum stability analysis of selected mini-hepcidins. Human male serum was centrifuged at 20,000 g for 10 minutes and the clear part of the supernatant was then diluted to 25% (v/v) with Milli-Q® water and incubated at 37°C for 15 minutes. Each peptide sample was dissolved in triplicate at a final concentration of 20 μM in pre-incubated 25% serum, or Milli-Q® water as a control. Aliquots of 100 μL were removed at different time points and quenched with 100 μL of 15% trichloroacetic acid (TCA), followed by incubation on ice for 30 min and centrifugation at 14,000 g for 5 minutes. Supernatant was then removed and transferred into vials for liquid chromatography mass spectrometry (LC-MS) analysis (50 μL injection and multiple reaction monitoring (MRM) protocol). Error bars indicate SEM, n=3. Comparison between Hep9, ***2***, Hep9[Ser^1, MeThr^2, MeIle^8], ***7***, and cHep9, ***9***.
